# Total versus partial adrenalectomy in bilateral pheochromocytoma – a systematic review and meta-analysis

**DOI:** 10.3389/fendo.2023.1127676

**Published:** 2023-03-14

**Authors:** Karolina Zawadzka, Piotr Tylec, Piotr Małczak, Piotr Major, Michał Pędziwiatr, Magdalena Pisarska-Adamczyk

**Affiliations:** ^1^ 2nd Department of General Surgery, Jagiellonian University Medical College, Kraków, Poland; ^2^ Jagiellonian University Medical College, Doctoral School of Medical and Health Sciences, Kraków, Poland; ^3^ Department of Medical Education, Jagiellonian University Medical College, Kraków, Poland

**Keywords:** pheochromocytoma, adrenalectomy, cortical-sparing, bilateral, partial, systematic review

## Abstract

**Background:**

In patients with bilateral pheochromocytoma, partial adrenalectomy offers the chance to preserve adrenal function and avoid the need for lifelong steroid supplementation. However, the risk of tumour recurrence raises questions about this procedure. The aim of our study was to compare partial and total adrenalectomy in bilateral pheochromocytoma through a systematic review with meta-analysis.

**Methods:**

A systematic search was carried out using databases (MEDLINE, EMBASE, Scopus, Web of Science, CENTRAL) and registers of clinical trials (ClinicalTrials.gov, European Trials Register, WHO International Trials Registry Platform). This meta-analysis included studies up to July 2022 without language restrictions. A random effects model meta-analysis was performed to assess the risk of tumor recurrence, steroid dependence and morbidity in these patients.

**Results:**

Twenty-five studies were included in the analysis involving 1444 patients. The relative risk (RR) of loss of adrenal hormone function during follow-up and the need for steroid therapy was 0.32 in patients after partial adrenalectomy: RR 0.32, 95% Confidence Interval (CI): 0.26-0.38, P < 0.00001, I2 = 21%. Patients undergoing partial adrenalectomy had a lower odds ratio (OR) for developing acute adrenal crisis: OR 0.3, 95% CI: 0.1-0.91, P=0.03, I2 = 0%. Partial adrenalectomy was associated with a higher risk of recurrence than total adrenalectomy: OR 3.72, 95% CI: 1.54-8.96, P=0.003, I2 = 28%.

**Conclusion:**

Partial adrenalectomy for bilateral pheochromocytoma is a treatment that offers a chance of preserving adrenal hormonal function, but is associated with a higher risk of local tumor recurrence. There was no difference for the risk of metastasis and in overall mortality among the group with bilateral pheochromocytomas undergoing total or partial adrenalectomy.

This study is in line with PRISMA (Preferred Reporting Items for Systematic Reviews and Meta-Analyses) and AMSTAR (A Measurement Tool to Assess Systematic Reviews) Guidelines (10, 11).

**Systematic review registration:**

https://osf.io/zx3se.

## 1 Introduction

Pheochromocytomas are catecholamine secreting tumors of adrenal medulla and together with paragangliomas, extra-adrenal tumors of sympathetic system, are referred to as paragangliomas and pheochromocytomas (PPGL) (1). The standard treatment for pheochromocytoma is adrenalectomy. However, for bilateral pheochromocytoma tumours, possible options include removing both adrenal glands or attempting to remove only the tumours to preserve adrenal hormonal function (2, 3).

Bilateral total adrenalectomy (TA) is accompanied by severe burdens as it causes permanent adrenal insufficiency, requiring lifelong corticosteroid replacement therapy. This can lead to undertreatment with corticoids, even resulting in a life-threatening Addisonian crisis. Recent studies also suggest that lifelong use of steroid hormone replacement is associated with reduced quality of life for patients and higher mortality (4).

Partial adrenalectomy (PA), also known as cortical-sparing adrenalectomy, is a technique in which surgeon preserves a small vascularized tumor-free part of adrenal tissue which should maintain adrenal hormones secretion (5, 6). Some studies suggest that the incidence of perioperative complications is comparable between total and partial adrenalectomy (7). However, concerns have been raised about the higher incidence of local relapses and the development of metastasis after PA, as adrenal cortex-sparing surgery inherently carries risk that some of the core tissue may not be removed (8, 9).

Endocrine Society Clinical Practical Guidelines propound partial adrenalectomy for bilateral pheochromocytoma. Notwithstanding, the level of evidence for this recommendation is low (1). Strong evidence favoring cortical sparing adrenalectomy in bilateral pheochromocytoma is lacking – there has been no systematic review with meta-analysis comparing these two interventions. The benefits of PA must be carefully weighed against the possible risks and therefore, the purpose of our study was to compare the results of total versus partial bilateral adrenalectomy in regards to recurrence, steroid dependence, development of metastatic pheochromocytoma, incidence of acute adrenal crisis, mortality and morbidity.

## 2 Methods

### 2.1 Search strategy

To identify studies comparing total versus partial (cortical-sparing) adrenalectomy for bilateral pheochromocytoma, we performed comprehensive literature search of the electronic databases (MEDLINE, EMBASE, Scopus, Web of Science, CENTRAL) as well as registers of clinical trials (ClinicalTrials.gov, European Trials Register, WHO International Trials Registry Platform).

Search strategy was based on the following terms: “pheochromocytoma” OR paraganglioma” OR PPGL” AND “partial” OR “subtotal” OR “cortical-sparing” OR “adrenal-sparing”. The full search strategies for all databases and registers are available in **Supplementary Table 1**. There were no language limitations in the search. In addition, reference lists of all included studies and review articles were assessed for additional references. All searches were performed on July 2022.

The protocol was preregistered in the International Open Science Framework Registry with an identification address: https://osf.io/zx3se/. This study is in line with PRISMA (Preferred Reporting Items for Systematic Reviews and Meta-Analyses) and AMSTAR (A Measurement Tool to Assess Systematic Reviews) Guidelines (10, 11).

### 2.2 Study selection and data extraction

Studies comparing total adrenalectomy with cortical-sparing adrenalectomy in adults with bilateral pheochromocytoma were considered for inclusion in this review. A search was conducted in July 2022 and there were no language limitations. We applied no restrictions on length of follow-up after surgery. To get a complete picture of the efficacy and harms of both types of surgery, we decided to include both randomised trials and non-randomised studies of interventions. The exclusion criteria were as follows (1): studies describing only a single intervention (total either partial adrenalectomy) (2), studies without primary or sufficient data: reviews, guidelines, conference abstracts, letters, comments (3), studies that included only patients with metastatic tumors. We considered a “partial adrenalectomy” to be both a bilateral partial adrenalectomy and a complete removal of one adrenal gland combined with a partial removal of the other adrenal gland.

**Figure 1:**
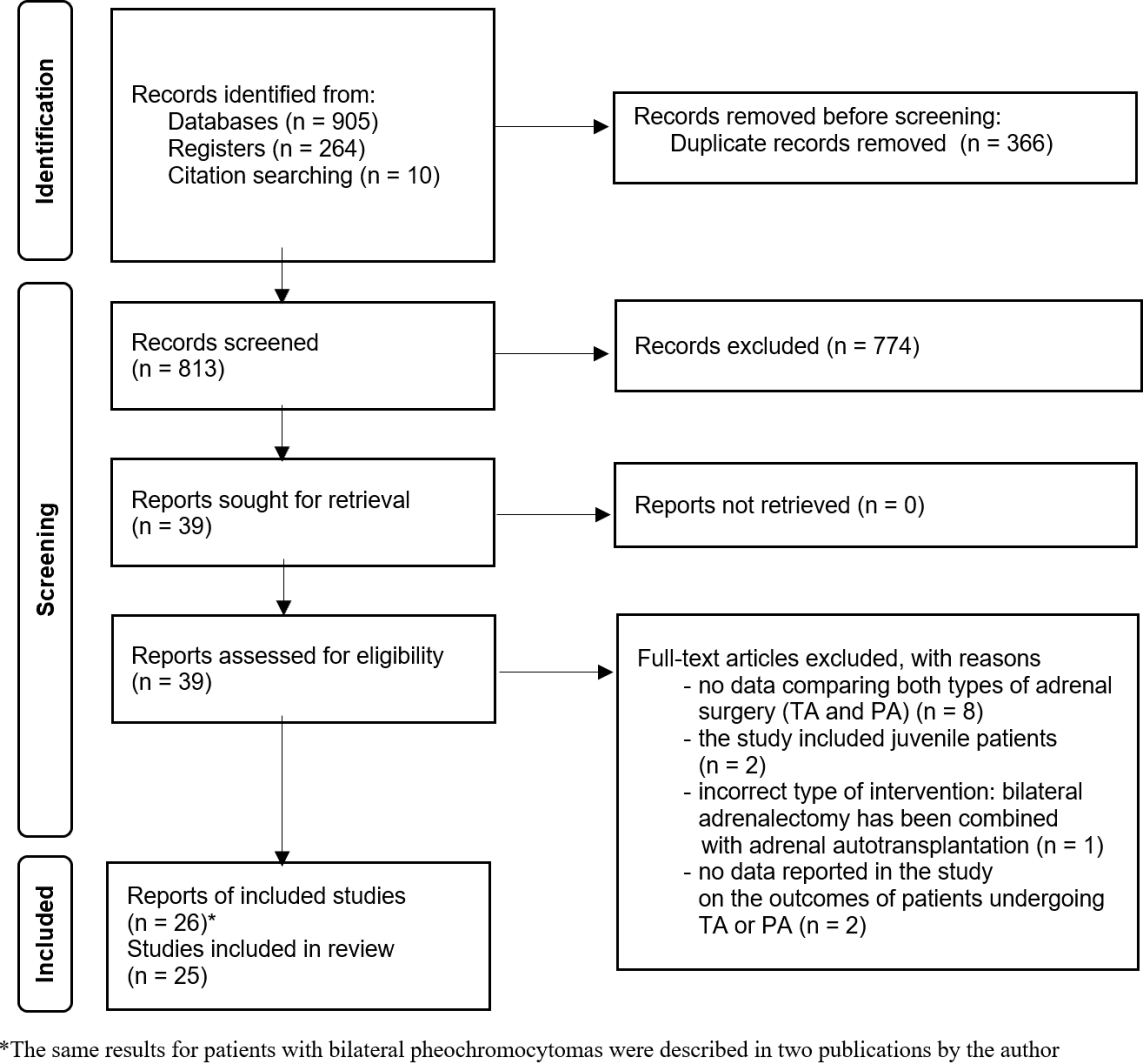
PRISMA 2020 flow diagram of the search strategy and study selection.

The titles and abstracts of the articles were screened independently by two reviewers (KZ, PT). Full texts were assessed by KZ and PT to determine their potential for inclusion, and the reasons for excluding ineligible studies were noted. In case of disagreements, discussion was made to reach consensus within the group. If required, we consulted a third review author (MPA). We also identified and excluded duplicates of the same study. The process of identifying relevant studies is summarized in PRISMA flow diagram (**Figure 1**).

The following study characteristics were extracted independently by two review authors (KZ, PT) from the included studies: first author, year of publication, study location, type of study, duration of follow-up, number, age and sex of both all participants and in TA versus PA group, the occurrence of genetic syndromes in included patients with PPGL, the incidence of synchronous and metachronous PPGL, loss to follow-up, data on intervention and comparison, and outcomes of interest, described in the paragraph below. The authors of the studies were contacted *via* e-mail if there was a need for additional data or necessary explanations (2, 12–14).

### 2.3 Outcome measures

Primary outcomes included: risk of pheochromocytoma recurrence and steroid dependence, defined as the need for lifelong glucocorticoid substitution. The secondary outcomes of interest were: time to recurrence after surgery, development of metastatic pheochromocytoma, incidence of adrenal crisis, morbidity, overall mortality and pheochromocytoma-specific mortality (caused by an adrenal crisis or metastatic pheochromocytoma).

### 2.4 Assessment of quality

The risk of bias in the included studies was assessed using The Risk Of Bias In Non-randomized Studies of Interventions (ROBINS-I) tool (**Supplementary Table 2**) (15). This was done in pairs by two review authors (KZ, PT). Any disagreements were resolved by discussion or by involving another review author (MPA). This tool consists of seven domains (bias due to confounding, bias in selection of participants into the study, bias in classification of interventions, bias due to deviations from intended interventions, bias due to missing data, bias in measurement of outcomes, bias in selection of the reported result and overall bias).

### 2.5 Statistical analysis

A meta-analysis was conducted using Review Manager (RevMan 5.4). For dichotomous outcomes, the risk ratio (RR) and odds ratio (OR) with 95% confidence intervals (CIs) were calculated. For continuous variable (time to recurrence), the weighted mean difference (WMD) with 95% CI was provided. In all analyses, the random-effects model was used to pool the result, as heterogeneity existed between the included studies. The forest plots were used to present the statistical results of the meta-analysis. For estimation of mean and standard deviation from median, interquartile range, minimum and maximum, we used methods described by Luo et al. and Wan et al. (16, 17).

Heterogeneity between the studies was assessed with the Cochrane Q test and the I2 statistic. We followed the interpretation in the Cochrane Handbook and considered I2 < 40% and I2 > 75% as corresponding to low and high heterogeneity, respectively (18). Statistically significant difference was observed at a two-tailed 0.05 level for the hypothesis and with 0.10 for heterogeneity testing.

For analyses which included at least 10 studies, Egger’s tests was performed to identify possible small-trial biases, such as publication bias (19). A P < 0.10 was considered statistically significant. Leave-one-out sensitivity analyses were conducted to assess the robustness of the pooled results. In addition, we contacted authors who had two publications that met the inclusion criterion for our meta-analysis to determine whether there was any overlap between the patient populations of the two publications.

## 3 Results

The literature search yielded 1169 records, including 905 studies and 264 research registers. Following deduplication, 813 records were screened based on titles and abstracts. Thirty nine trials were selected for full-text analysis and we identified twenty six records. Janetschek et al. described in two publications the results of 4 adult patients who underwent partial bilateral adrenalectomy and 1 patient who underwent bilateral total adrenalectomy (20, 21). For further analysis, we included a study issued in 1998 because it contained more patient data, and twenty-five observational studies were finally qualified for qualitative and quantitative analysis. We found no randomised study comparing total adrenalectomy and partial adrenalectomy for bilateral pheochromocytomas.

The studies involved 1444 subjects (826 patients treated by bilateral total adrenalectomy and 618 patients undergoing partial adrenalectomy). **Figure 1** presents the flowchart of the search process.

The focus of five studies (21–25) was laparoscopic surgery for all pheochromocytoma tumors and among them we extracted data on a group of patients with bilateral PPGL.

Most of the studies gained insight into a group of patients with hereditary PPGL, most of whom were bilateral pheochromocytomas (9, 12–14, 26–33).

Seven studies analyzed only patients with bilateral PPGL, both with sporadic and hereditary tumors (2, 3, 7, 34–37). Two authors analyzed data of patients with bilateral PPGL, all of which had a genetic background (36, 38).

Eight studies analyzed only RET mutation carriers (9, 12, 13, 27, 29, 31, 33, 38) and two studies focused on patients with von Hippel-Lindau disease (26, 30).

The contribution of heredity among the remaining studies on bilateral PPGL ranged from 50 to 96% (3, 34). RET was the most frequently mutated gene in hereditary PPGL (2, 3, 7, 9, 12–14, 21, 23, 27–29, 31–38). Two studies did not provide information on the genetic background of bilateral tumors (22, 24)

The characteristics of the included trials are summarised in **Table 1**.

**Table 1:** Summary of included studies.

First author, year (country)	Genetic background (%, no. of patients)	No. of synchronous/metachronous PPGL	No. of patients TA/PA	Mean duration of follow-up (years)	Overall risk of bias (ROBINS-I tool)	COI	Funding
Asari 2006, (Austria)	100% (10 MEN2A)	5/5	9/1	6.8	Serious risk	No	NR
Baghai 2002, (United States)	100% (4 VHL)	4/0	2/2	6.8	Serious risk	NR	NR
Castillo 2007, (Chile)	66.67% (2 VHL, 1 MEN2A, 1 FP)	6/0	4/2	1.3	Critical risk	NR	NR
Castillo 2011, (Chile)	50% (1 VHL, 2 MEN2, 1 FP)	8/0	5/3	7.6	Serious risk	NR	NR
Castinetti 2014 (Multicenter study)	100% (345 MEN2)	250/95	257/82	13	Serious risk	No	Yes
Castinetti 2019 (Multicenter study)	100% (111 MEN2B)^1^	79/32^	69/26	NM	Serious risk	No	No
Goretzki 1996 (Austria)	100% (7 MEN2)	7/0	4/3	4.9	Serious risk	NR	NR
Grubbs 2013 (United States)	76% (36 MEN2A, 5 MEN2B, 4 VHL, 1 NF1, 1 MEN1)	47/15	25/39	10.9	Serious risk	NR	NR
Iihara 2003 (Japan)	86% (5 MEN2A, 1 VHL)	7/0	4/3	1.3	Serious risk	NR	NR
Inabnet 2000 (United States)	100% (14 MEN2A, 7 VHL, 2 NF1, 1 FP)^2^	NM	5/5	7	Serious risk	NR	NR
Janetschek 1998 (Germany)	100% (1 MEN2A, 1 MEN2B, 3 VHL)	4/1	¼	1.1	Serious risk	NR	NR
Jansson 2006 (Sweden)	100% (2 MEN2A, 4 MEN2B, 3 NF1)	6/3	3/6	13	Serious risk	NR	NR
Kittah 2020 (United States)	80% (40 MEN2A, 18 VHL, 9 MEN2B, 8 NF1)	75/19	59/18	10.6	Serious risk	Yes	No
Lee 1996 (United States)	100% (10 MEN2A, 2 MEN2B, 3 VHL)	12/3	1/14	12.4	Critical risk	NR	NR
Neumann 2019 (Multicenter study)	96% (282 MEN2, 184 VHL, 39 other genes)	401/224	301/324	12.2	Serious risk	Yes	Yes
Nockel 2018 (United States)	RET, VHL, NF1, SDHA-D, MAX, and FH mutations carriers^3^	12/2	4/10	NM	Critical risk	NR	NR
Pugliese 2008 (Italy)	NM	NM	1/1	10 days	Critical risk	No	NR
Qi 2013 (China)	100% (17 MEN2A)	14/3	7/9	6	Serious risk	NR	NR
Rajan 2016 (India)	100% (8 MEN2)	7/1	6/2	3.6	Serious risk	NR	NR
Sanford 2021 (United States)	100% (21 VHL)	19/2	8/13	16.7	Serious risk	No	NR
Scholten 2011 (The Netherlands)	100% (24 MEN2)	NM	22/2	15.4	Serious risk	NR	NR
Simforoosh 2020 (Iran)	NM	6/0	4/2	5.4	Serious risk	No	NR
Van Heerden 1984 (United States)	100% (14 MEN2A, 3 MEN2B)^4^	NM	8/3	10.8	Critical risk	NR	NR
Walz 2006 (Germany)	87% (6 MEN2A, 2 MEN2B, 8 VHL, 3 SDHD, 1 NF1)	15/8	1/22	3.8	Serious risk	NR	NR
Yip 2004 (United States)	89% (26 MEN2A, 4 MEN2B, 4 VHL)	31/7	16/22	8.25	Serious risk	No	Yes

COI, conflict of interest; NR, not reported.

1 From 111 patients with bilateral pheochromocytoma, 95 of them had TA or PA adrenalectomy.

2 Of the 24 patients enrolled in the study, 14 underwent heterotopic autotransplantation of cortical tissue, 5 underwent total bilateral adrenalectomy, and 5 underwent cortical-sparing adrenalectomy.

3 It was not possible to extract the number of patients with bilateral pheochromocytoma with specific mutations.

4 Of the 17 patients enrolled in the study, 11 had bilateral pheo, 5 had unilateral pheochromocytoma and contralateral adrenal medullary hyperplasia (AMH) and 1 had bilateral AMH.

### 3.1 Risk of recurrence

**Figure 2:**
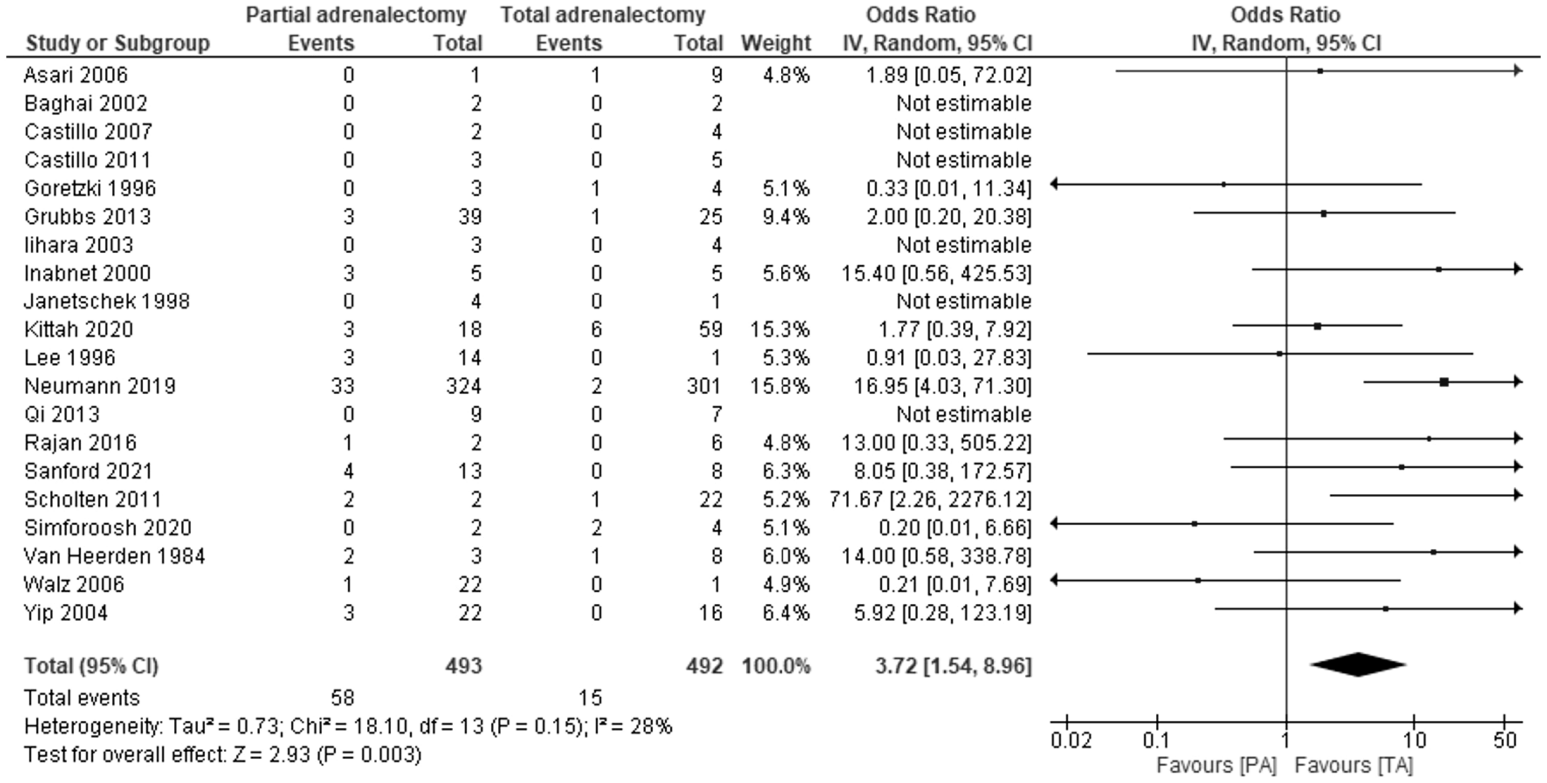
Pooled estimates of risk of recurrence comparing TA vs PA. CI, confidence interval; df, degrees of freedom.

Data on recurrence of PPGL after total and adrenal-sparing adrenalectomy have been reported in 20 studies with 985 patients. The results of 14 studies, with the mean duration of follow-up ranged from 3.6 years in the Rajan 2016 study to 16.7 years in the Sanford 2021 study, showed PA is associated with a higher risk of recurrence than TA – OR 3.72, 95% CI: 1.54-8.96, P=0.003, I2 = 28% (**Figure 2**). Noticeably, in 6 studies, during the follow-up period, no recurrences of pheochromocytoma were noted in both TA and PA groups. Egger’s regression test showed no significant asymmetry (P=0.219).

### 3.2 Steroid dependence

Twenty-two studies with 1404 patients reported data on this outcome. All patients undergoing total bilateral adrenalectomy were steroid-dependent, while approximately two-thirds did not require steroid supplementation after PA: RR 0.32, 95% CI: 0.26-0.38, P<0.00001, I2 = 21%.

### 3.3 Development of Addisonian crisis

**Figure 3:**
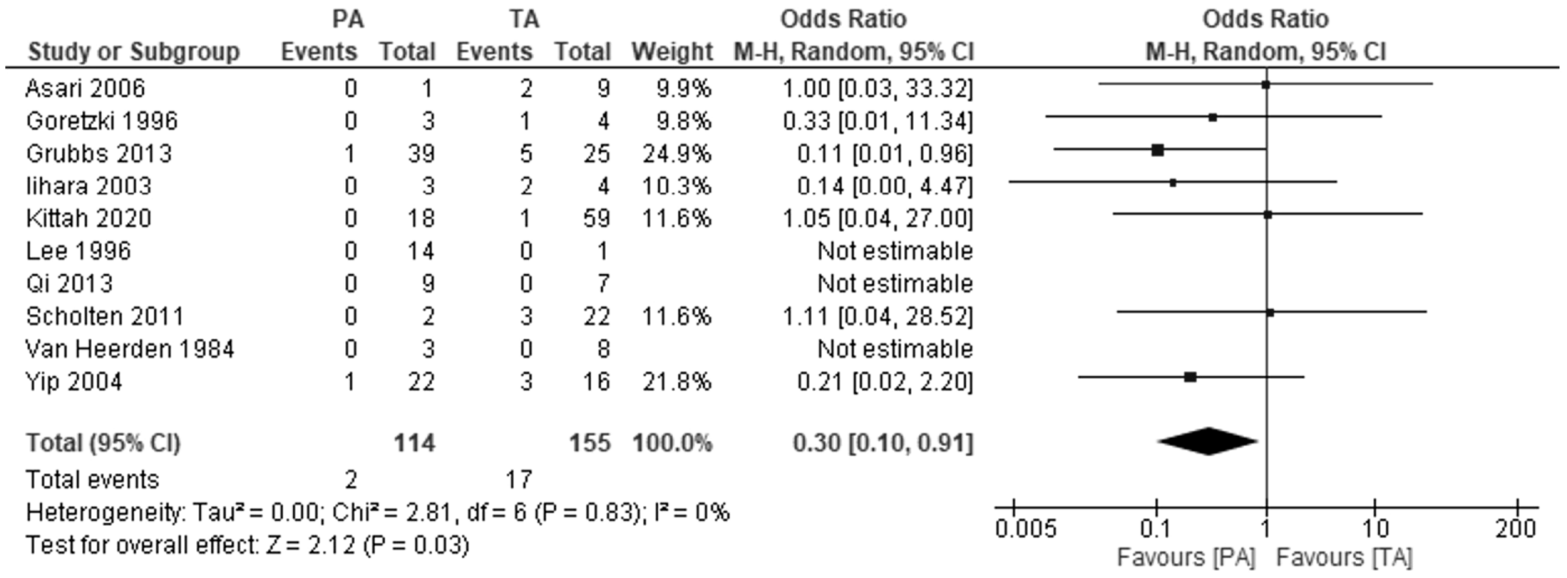
Pooled estimates of risk of development of addisonian crisis comparing TA vs PA. CI, confidence interval; df, degrees of freedom.

Ten studies investigated the risk of acute adrenal insufficiency. Patients undergoing partial adrenalectomy had almost three times lower risk of developing acute adrenal insufficiency: OR 0.3, 95% CI: 0.1−0.91, P=0.03, I2 = 0% (**Figure 3**). In three studies, no adrenal crisis was noted in both groups during the follow-up period.

### 3.4 Development of metastatic pheochromocytoma

**Figure 4:**
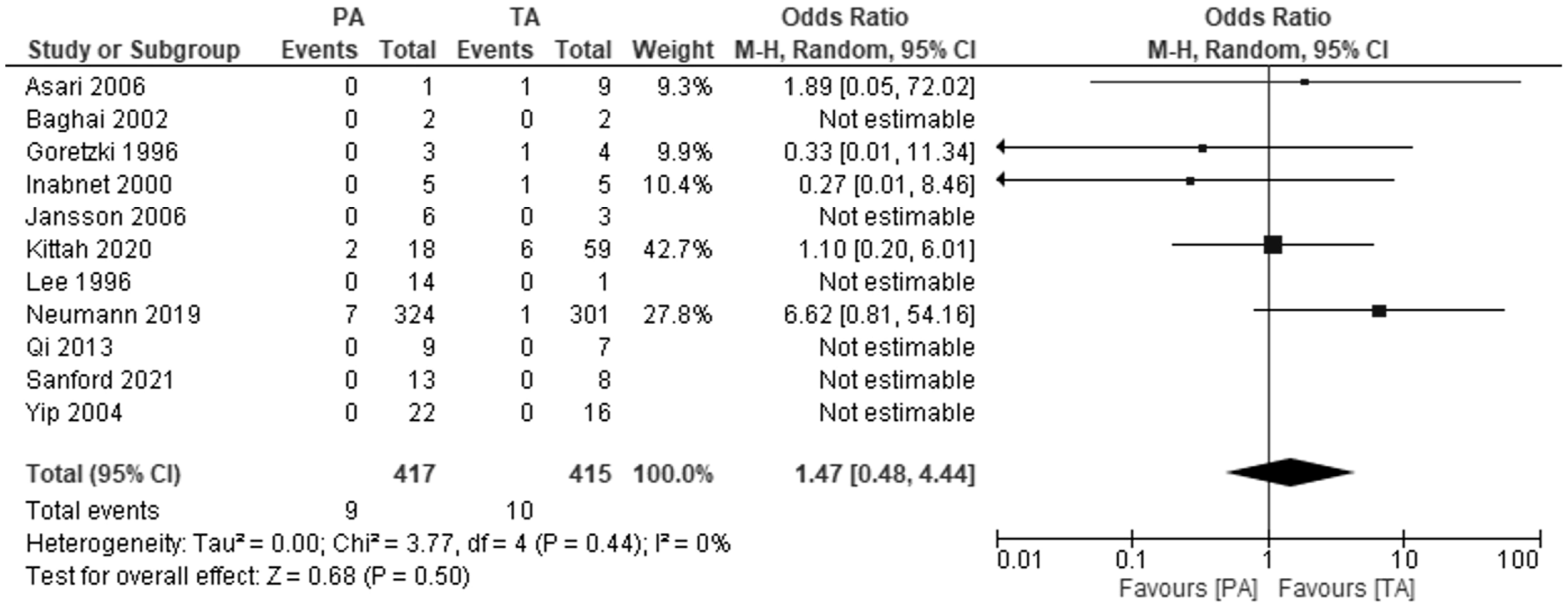
Pooled estimates of risk of development of metastatic pheochromocytoma comparing TA vs PA. CI, confidence interval; df, degrees of freedom.

In five studies comparing the development of metastatic pheochromocytoma in patients after TA and PA, we found no difference between the two groups: OR 1.47, 95%CI: 0.48-4.44), P=0.5, I2 = 0%. The mean duration of follow-up ranged from 4.9 years in the Goretzki 1996 study to 12.2 years in the Neumann 2019 study. Six studies reported that during the in the mean follow-up from 6 to 16.7 years, no patients, regardless of the group, developed metastases (**Figure 4**).

### 3.5 Overall mortality

**Figure 5:**
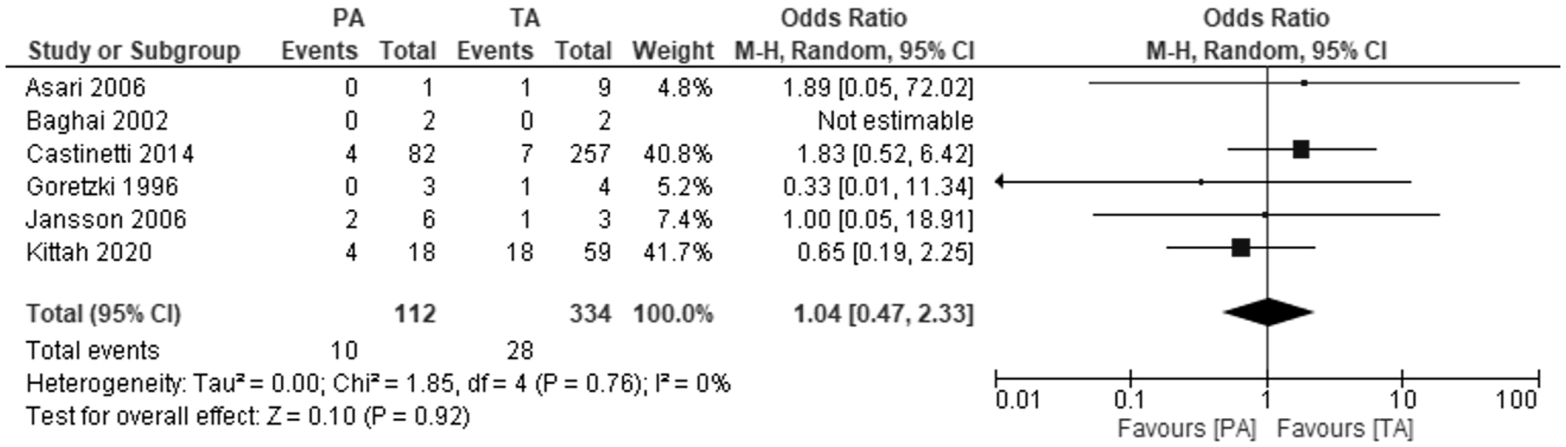
Pooled estimates of risk of overall mortality comparing TA vs PA. CI, confidence interval; df, degrees of freedom.

Six studies investigated overall mortality rate (**Figure 5**). The meta-analysis showed no differences between PA and TA patients at follow-up from 4.9 to 13 years: OR 1.04, 95% CI: 0.47-2.33, P=0.92, I2 = 0%.

### 3.6 Pheochromocytoma-specific mortality

Five studies reported pheochromocytoma-specific mortality. Two studies reported no pheochromocytoma-related deaths during 6.8 and 13 years of follow-up, respectively. The other three studies found no difference between groups in pheochromocytoma-related mortality at follow-up from 4.9 to 10.6 years - OR: 0.54, 95% CI: 0.08-3.72, P=0.53 (**Supplementary Figure 1**). There was no heterogeneity between studies, I2 = 0%.

### 3.7 Time to recurrence

Only one study provided data on time to relapse in both PA and TA groups, and there was no difference between treatment groups: OR 7.22, 95% CI: -4.39 to 18.83, P=0.22.

### 3.8 Publication bias and quality of evidence

All studies were independently assessed by two researchers (KZ, PT) using the ROBINS-I tool. The assessment of the risk of bias for each study is presented in **Supplementary Table 2**. Most of the studies included in the meta-analysis were ranked with a significant risk of bias. This is due to the fact authors did not use an appropriate analysis method that controlled for confounding. A significant proportion of the studies described the outcomes of patients with the genetic basis of PPGL, and their purpose was not focused on comparing PA or TA (9,14, 21–26, 28, 29, 32, 33, 35, 36). Thus, the balance between comparator groups at baseline was difficult to assess due to limited characteristics of a study population.

All the studies included in the meta-analysis were non-randomized retrospective ones, therefore it should be taken into account that the decisions to assign patients to TA or PA were made by different surgeons, whose clinical experience in adrenal cortex-sparing surgery could differ.

Egger’s test revealed no evidence of publication bias for the risk of PPGL recurrence. For secondary outcomes, we were unable to explore the small-study effect due to the low number of studies.

### 3.9 Sensitivity analysis

As we mentioned previously, the patient populations from Janetschek’s two publications overlapped, so we included one of his two publications in the meta-analysis. In the case of the Castinetti publication, the author declared that the patient populations differ in the two publications (one focuses on patients with MEN2A syndrome and the other on patients with MEN2B). We did not get a response from Castillo, so we performed a sensitivity analysis in the meta-analysis on the risk of recurrence in which his results were included. Despite the removal of the Castillo 2007 study and then Castillo 2011 the results of the meta-analysis remained unchanged. We also tested the possible impact of individual studies, so we applied a sensitivity analysis that eliminated one included study at a time (**Supplementary Figures 2A–E**). The results revealed that changes in the pooled effect estimates were not evident, indicating that the results were robust.

## 4 Discussion

This systematic review and meta-analysis of 25 retrospective studies on bilateral pheochromocytoma showed that tumor recurrence following partial adrenalectomy is considerably higher than after total adrenalectomy. Nevertheless, the risk of developing metastases, all-cause mortality, and pheochromocytoma-specific mortality do not differ in the total and partial adrenalectomy groups. The major advantage of partial adrenalectomy over complete removal of the adrenal glands is the reduced incidence of steroid dependence and hence the reduced risk of developing an adrenal crisis.

Currently, total adrenalectomy represents the most common choice for bilateral pheochromocytoma (35). Nevertheless, for the last years, the concept of preserving cortical cortex through partial adrenalectomy has been increasingly proposed as an alternative surgical option in pheochromocytoma (3, 12, 13). PA offers a chance to avoid the complete dependence on steroids which is inextricably linked with bilateral adrenal removal (12). The necessity of lifelong steroid supplementation is burdensome for patients in terms of matching the appropriate dose. Both overdose and a deficiency of steroids have debilitating effects, from the symptoms of Cushing’s syndrome to an excess of steroids to the risk of developing an adrenal crisis if the dose is too low. The above complications contribute to significant depreciation in quality of life in these patients (39). One of the most dangerous complications in patients with adrenal insufficiency, which is adrenal crisis, is associated with an approximately 6% risk of death. Moreover, it is common even in patients who are well educated about the disease and glucocorticoid adjustments for stressful events (40). The results of our meta-analysis revealed that in this regard, partial adrenalectomy offers a clear advantage, as patients after PA are three times less likely to develop Addison’s crisis. However, it is also important to note that despite advances in adrenal-sparing techniques, these methods are still not ideal. While some part of the adrenal cortex remains, over time some patients after PA also lose the ability to endogenously synthesize adrenal hormones. The pooled results of our meta-analysis showed that in 28% of patients, the treatment to preserve adrenal function failed and they became steroid dependent. There were considerable differences between the studies here - some authors did not report any cases of steroid dependence in PA patients, and in those who experienced loss of function, the proportion ranged from 4 to 44% (2, 23). This suggests that there may be significant discrepancies in the effectiveness of adrenal-sparing surgical techniques between different centers and perhaps this type of surgery should only be performed in selected, highly specialized centers for the treatment of pheochromocytomas, especially bilateral ones.

Preservation of residual cortical function, offering a chance to avoid adrenal insufficiency is weighed against the increased risk of local recurrence. While in the 1980s even 2 out of 3 patients undergoing sparing surgery experienced recurrence, recent studies on large populations show that only 5-17% of patients undergoing sparing surgery will experience relapse (2, 3, 12, 33). Despite this progress, the results of our meta-analysis showed that the risk of recurrence is still more than three times higher in PA patients than in TA patients. The problem of oncological completeness arises from the fact that it is extremely difficult to ensure that there is no residual adrenal medulla tissue (5).

New light on the matter is shed by the use of an indocyanine green fluorescence, which not only allows differentiation of the adrenal gland from the surrounding retroperitoneal structures, but also can be used to identify the border between tumor and adrenal cortex during partial adrenalectomy. Adrenal cortex-sparing surgeries performed on several patients have shown that this technology can be immensely useful for precise tumor resection by identifying hypofluorescent pheochromocytoma and hyperfluorescent cortical tissue (41, 42).

Our systematic review with meta-analysis is not devoid of limitations. The review is based on an analysis of observational studies only, so decisions to allocate patients to total or partial adrenalectomy surgery were made by local surgeons based on a number of different factors. Additionally, none of the studies took into account confounding factors. Some of the studies have focused on other aspects of treating bilateral phaeochromocytomas, including the descriptive characteristics of the outcomes of patients with specific genetic mutations associated with PPGL (13, 28, 30, 38, 39). Thus, we often do not know if baseline data were comparable in the total and partial adrenalectomy groups. We are also unable to determine whether the important co-interventions were balanced across intervention groups. These factors contribute to the low quality of the analysed research. The included retrospective studies, which did not take into account confounding factors, had to be classified as having serious bias according to the ROBINS-I tool. Another limitation of the meta-analysis is the fact that partial adrenalectomy was used as a partial surgery on either side or the tumor was removed on one side and the adrenal gland was completely removed on the other side. It was not possible to extract enough data from studies to perform a separate subgroup analysis for both of the above situations. An important fact influencing the bias of the included studies is the duration of follow-up, which ranged from approximately one year to seventeen years (21, 30, 35). This undoubtedly influenced the key outcomes such as the recurrence rate, but also the loss of adrenal function and steroid dependence or the occurrence of an adrenal crisis. In addition, studies with zero events were not included in the forest plots since they are statistically omitted when pooling relative effects. Importantly, several studies reported no pheochromocytoma recurrence, acute adrenal insufficiency, metastatic development, and pheochromocytoma-specific mortality in both total and partial adrenalectomy groups during the follow-up.

Considering the very rare occurrence of bilateral pheochromocytoma, which, according to the latest data, occurs in less than 10% of pheochromocytoma patients, it would be very challenging to conduct a prospective, randomized trial (2). Also, recent large multicenter studies, although they have made valuable contributions to the topic of the effects of cortical-sparing treatment, are still not without bias because of the different local experiences with adrenal surgery and the lack of clear indications of which patients were referred for partial adrenalectomy and which for complete removal of both adrenal glands (3, 12).

## 5 Conclusion

In conclusion, partial adrenalectomy is a feasible option for bilateral pheochromocytoma, offering a greater chance of avoiding lifelong steroid dependence and associated complications. While partial adrenalectomy carries a higher risk of recurrence, it is not associated with an increased risk of metastasis and mortality in patients with pheochromocytoma. The development of new imaging techniques and training of adrenal surgeons should be pursued to facilitate accurate differentiation of cortical from medullary adrenal tissue and thereby contribute to a lower recurrence rate and reduced steroid dependency rate after partial adrenalectomy.

## Data availability statement

The original contributions presented in the study are included in the article/**Supplementary Material**. Further inquiries can be directed to the corresponding author.

## Author contributions

KZ conceived and designed the study. KZ, PT, PMaj and PMał performed studies search. KZ and PT extracted information. KZ and PT drafted the manuscript, which was revised by PMaj, MP, and MP-A. All authors contributed to the article and approved the submitted version.
