# Supplementary material for: Total versus partial adrenalectomy in bilateral pheochromocytoma – a systematic review and meta-analysis
Source: Front Endocrinol (Lausanne). 2023 Mar 14;14:1127676. doi: 10.3389/fendo.2023.1127676 (PMC10043479; doi:10.3389/fendo.2023.1127676)

Supplementary Material

Total versus partial adrenalectomy in bilateral pheochromocytoma – a systematic review and meta-analysis

**Karolina Zawadzka^1,2^, Piotr Tylec^1^, Piotr Małczak^1^, Piotr Major^1^, Michał Pędziwiatr^1^, Magdalena Pisarska-Adamczyk^3*^**

*** Correspondence:** Magdalena Pisarska-Adamczyk MD PhD, Email: [magdalena.pisarska@uj.edu.pl](mailto:magdalena.pisarska@uj.edu.pl)

**Supplemental Tables**

**Supplementary Table 1** Full search strategies for MEDLINE, Embase, Web of Science, Scopus and CENTRAL

**Supplementary Table 2** Risk-of-bias assessment of the included studies using the The Risk Of Bias In Non-randomized Studies of Interventions (ROBINS-I) tool

**Supplemental Figures**

**Supplementary Figure 1** Pooled estimates of pheochromocytoma-specific mortality comparing TA vs PA

**Supplementary Figure 2** Results of sensitivity analyses with omission of one study at a time

**Supplementary Table 1** Full search strategies for MEDLINE, Embase, Web of Science, Scopus and CENTRAL

| **MEDLINE PubMed** |
| --- |
| #10 (#9 NOT (animals [mh] NOT humans [mh])) |
| #9 (#1 AND #4 AND #5 AND #8) |
| #8 (#6 OR #7 ) |
| #7 (adrenalectomy* [tiab] OR "Adrenalectomy"[Mesh]) |
| #6 (((pheochromocytom* [tiab] OR phaeochromocytom* [tiab] OR paragangli* [tiab] OR PPGL [tiab] OR adrenal* [tiab] OR “adrenal gland” [tiab]) AND (remov* [tiab] OR resect* [tiab] OR surg* [tiab] OR operat* [tiab])) OR "Pheochromocytoma/surgery"[Mesh]) |
| #5 (bilateral* [tiab] OR heredita* [tiab] OR MEN2* [tiab] OR RET [tiab] OR VHL [tiab] OR NF1 [tiab] OR SDHA* [tiab] OR SDHB* [tiab] OR SDHC* [tiab] OR SDHD*[tiab] OR SDHAF2* [tiab] OR MAX [tiab] OR EPAS1 [tiab] OR FH [tiab] OR TMEM127 [tiab] OR HIF2A [tiab] OR HRAS [tiab] OR KIF1B* [tiab] OR PHD2 [tiab] OR neurofibromato* [tiab] OR „Neurofibromatosis 1” [Mesh] OR „von Recklinghausen's disease” [tiab] OR „Multiple endocrine neoplasia” [Mesh] OR „Multiple endocrine neoplasia” [tiab] OR „von Hippel-Lindau Disease” [Mesh] OR von Hippel-Lindau* [tiab]) |
| #4 (#2 OR #3) |
| #3 ((adren* [tiab] OR organ* [tiab] OR cortic* [tiab]) AND (preserv* [tiab] OR sparing* [tiab])) |
| #2 (partial* [tiab] OR subtotal* [tiab]) |
| #1 (pheochromocytom* [tiab] OR phaeochromocytom* [tiab] OR pheochromoblastom* [tiab] OR PCC [tiab] OR "Adrenal Gland Neoplasms" [tiab] OR “Adrenal Gland Tumor” [tiab] OR “Adrenal Medulla Tumor” [tiab] OR "Adrenal Gland Neoplasms"[Mesh] OR "Paraganglioma"[Mesh] OR paragangli* [tiab] OR PPGL* [tiab]) |
| **Embase** |
| #9 (#1 AND #4 AND #5 AND #8) |
| #8 (#6 OR #7 ) |
| #7 (adrenalectomy*:ti,ab,kw OR ‘Adrenalectomy’/exp) |
| #6 (((pheochromocytom*:ti,ab,kw OR phaeochromocytom*:ti,ab,kw OR paragangli*:ti,ab,kw OR PPGL*ti,ab,kw OR adrenal*:ti,ab,kw OR ‘adrenal gland’:ti,ab,kw) AND (remov*:ti,ab,kw OR resect*:ti,ab,kw OR surg*:ti,ab,kw OR operat*:ti,ab,kw)) OR ‘Pheochromocytoma/surgery’/exp) |
| #5 (bilateral*:ti,ab,kw OR heredita*:ti,ab,kw OR MEN2*:ti,ab,kw OR RET:ti,ab,kw OR VHL:ti,ab,kw OR NF1:ti,ab,kw OR SDHA*:ti,ab,kw OR SDHB*:ti,ab,kw OR SDHC*:ti,ab,kw OR SDHD*:ti,ab,kw OR SDHAF2*:ti,ab,kw OR MAX:ti,ab,kw OR EPAS1:ti,ab,kw OR FH:ti,ab,kw OR TMEM127:ti,ab,kw OR HIF2A:ti,ab,kw OR HRAS:ti,ab,kw OR KIF1B*:ti,ab,kw OR PHD2:ti,ab,kw OR neurofibromato*:ti,ab,kw OR ‘Neurofibromatosis 1’/exp OR ‘von Recklinghausen* disease’:ti,ab,kw OR ‘Multiple endocrine neoplasia’/exp OR ‘Multiple endocrine neoplasia’:ti,ab,kw OR ‘von Hippel-Lindau Disease’/exp OR von Hippel-Lindau*:ti,ab,kw) |
| #4 (#2 OR #3) |
| #3 ((adren*:ti,ab,kw OR organ*:ti,ab,kw OR cortic*:ti,ab,kw) AND (preserv*:ti,ab,kw OR sparing*:ti,ab,kw)) |
| #2 (partial*:ti,ab,kw OR subtotal*:ti,ab,kw) |
| #1 pheochromocytom*:ti,ab,kw OR phaeochromocytom*:ti,ab,kw OR pheochromoblastom*:ti,ab,kw OR PCC*:ti,ab,kw OR ‘Adrenal Gland Neoplasms’:ti,ab,kw OR ‘Adrenal Gland Tumor’:ti,ab,kw OR ‘Adrenal Medulla Tumor’:ti,ab,kw OR ‘Adrenal Gland Neoplasms’/exp OR ‘Paraganglioma’/exp OR paragangli*:ti,ab,kw OR PPGL*:ti,ab,kw |
| **Web of Science All Databases** |
| #9 (#1 AND #4 AND #5 AND #8) |
| #8 (#6 OR #7 ) |
| #7 TS = (adrenalectomy) |
| #6 TS = ((pheochromocytom* OR phaeochromocytom* OR paragangli* OR PPGL OR adrenal*) AND (remov* OR resect* OR surg* OR operat*)) |
| #5 TS = (bilateral*OR heredita* OR MEN2* OR RET OR VHL OR NF1 OR SDHA* OR SDHB* OR SDHC* OR SDHD* OR SDHAF2* OR MAX OR EPAS1 OR FH OR TMEM127 OR HIF2A OR HRAS OR KIF1B* OR PHD2 OR neurofibromato* OR von Recklinghausen* OR Multiple endocrine neoplasia OR von Hippel-Lindau*) |
| #4 (#2 OR #3) |
| #3 TS = ((adren* OR organ* OR cortic*) AND (preserv* OR sparing*)) |
| #2 TS = (partial* OR subtotal*) |
| #1 TS = ((pheochromocytom* OR phaeochromocytom* OR pheochromoblastom* OR “PCC” OR paragangli* OR PPGL OR "Adrenal Gland Neoplasm*" OR “Adrenal Gland Tumor” OR “Adrenal Medulla Tumor”) |
| **Scopus** |
| #13 (#1 AND #5 AND #6 AND #12) |
| #12 (#7 OR #8 OR #9 OR #10 OR #11) |
| #11 TITLE-ABS-KEY (adrenalectomy) |
| #10 TITLE-ABS-KEY ( pheochromocytom* ) AND TITLE-ABS-KEY ( operat * ) OR TITLE-ABS-KEY ( phaeochromocytom* ) AND TITLE-ABS-KEY ( operat * ) OR TITLE-ABS-KEY ( paragangli* ) AND TITLE-ABS-KEY ( operat * ) OR TITLE-ABS-KEY ( ppgl ) AND TITLE-ABS-KEY ( operat * ) OR TITLE-ABS-KEY ( adrenal* ) AND TITLE-ABS-KEY ( operat* ) |
| #9 TITLE-ABS-KEY ( pheochromocytom* ) AND TITLE-ABS-KEY ( surg * ) OR TITLE-ABS-KEY ( phaeochromocytom* ) AND TITLE-ABS-KEY ( surg * ) OR TITLE-ABS-KEY ( paragangli* ) AND TITLE-ABS-KEY ( surg * ) OR TITLE-ABS-KEY ( ppgl ) AND TITLE-ABS-KEY ( surg * ) OR TITLE-ABS-KEY ( adrenal* ) AND TITLE-ABS-KEY ( surg* ) |
| #8 TITLE-ABS-KEY ( pheochromocytom* ) AND TITLE-ABS-KEY ( resect * ) OR TITLE-ABS-KEY ( phaeochromocytom* ) AND TITLE-ABS-KEY ( resect * ) OR TITLE-ABS-KEY ( paragangli* ) AND TITLE-ABS-KEY ( resect * ) OR TITLE-ABS-KEY ( ppgl ) AND TITLE-ABS-KEY ( resect * ) OR TITLE-ABS-KEY ( adrenal* ) AND TITLE-ABS-KEY ( resect* ) |
| #7 TITLE-ABS-KEY ( pheochromocytom* ) AND TITLE-ABS-KEY ( remov* ) OR TITLE-ABS-KEY ( phaeochromocytom* ) AND TITLE-ABS-KEY ( remov* ) OR TITLE-ABS-KEY ( paragangli* ) AND TITLE-ABS-KEY ( remov* ) OR TITLE-ABS-KEY ( ppgl ) AND TITLE-ABS-KEY ( remov* ) OR TITLE-ABS-KEY ( adrenal* ) AND TITLE-ABS-KEY ( remov* ) |
| #6 TITLE-ABS-KEY (bilateral*) OR TITLE-ABS-KEY (heredita*) OR TITLE-ABS-KEY (MEN2*) OR TITLE-ABS-KEY (RET) OR TITLE-ABS-KEY (VHL) OR TITLE-ABS-KEY (NF1) OR TITLE-ABS-KEY (SDHA*) OR TITLE-ABS-KEY (SDHB*) OR TITLE-ABS-KEY (SDHC*) OR TITLE-ABS-KEY (SDHD*) OR TITLE-ABS-KEY (SDHAF2*) OR TITLE-ABS-KEY (MAX) OR TITLE-ABS-KEY (EPAS1) OR TITLE-ABS-KEY (FH) OR TITLE-ABS-KEY (TMEM127) OR TITLE-ABS-KEY (HIF2A) OR TITLE-ABS-KEY (HRAS) OR TITLE-ABS-KEY (KIF1B*) OR TITLE-ABS-KEY (PHD2) OR TITLE-ABS-KEY (neurofibromato*) OR TITLE-ABS-KEY (von Recklinghausen*) OR TITLE-ABS-KEY (Multiple endocrine neoplasia) OR TITLE-ABS-KEY (von Hippel-Lindau*) |
| #5 (#2 OR #3 OR #4) |
| #4 TITLE-ABS-KEY (adren*) AND TITLE-ABS-KEY (sparing*) OR TITLE-ABS-KEY (organ*) AND TITLE-ABS-KEY (sparing*) OR TITLE-ABS-KEY (cortic) AND TITLE-ABS-KEY (sparing*) |
| #3 TITLE-ABS-KEY (adren*) AND TITLE-ABS-KEY (preserv*) OR TITLE-ABS-KEY (organ*) AND TITLE-ABS-KEY (preserv*) OR TITLE-ABS-KEY (cortic*) AND TITLE-ABS-KEY (preserv*) |
| #2 TITLE-ABS-KEY (partial*) OR TITLE-ABS-KEY (subtotal*) |
| #1 TITLE-ABS-KEY (pheochromocytom*) OR TITLE-ABS-KEY (phaeochromocytom*) OR TITLE-ABS-KEY (pheochromoblastom*) OR TITLE-ABS-KEY (PCC) TITLE-ABS-KEY (paragangli*) OR TITLE-ABS-KEY (PPGL) OR TITLE-ABS-KEY ("Adrenal Gland Neoplasm*") OR TITLE-ABS-KEY ( "Adrenal Gland Tumor") OR TITLE-ABS-KEY ("Adrenal Medulla Tumor") |
| **CENTRAL (Cochrane Central Register of Controlled Trials)** |
| #9 (#1 AND #4 AND #5 AND #8) |
| #8 (#6 OR #7 ) |
| #7 (adrenalectomy*:ti,ab,kw OR [mh ”Adrenalectomy”]) |
| #6 ((pheochromocytom*:ti,ab,kw OR phaeochromocytom*:ti,ab,kw OR paragangli*:ti,ab,kw OR PPGL*ti,ab,kw OR adrenal*:ti,ab,kw OR ‘adrenal gland’:ti,ab,kw) AND (remov*:ti,ab,kw OR resect*:ti,ab,kw OR surg*:ti,ab,kw OR operat*:ti,ab,kw)) |
| #5 (bilateral*:ti,ab,kw OR heredita*:ti,ab,kw OR MEN2*:ti,ab,kw OR RET:ti,ab,kw OR VHL:ti,ab,kw OR NF1:ti,ab,kw OR SDHA*:ti,ab,kw OR SDHB*:ti,ab,kw OR SDHC*:ti,ab,kw OR SDHD*:ti,ab,kw OR SDHAF2*:ti,ab,kw OR MAX:ti,ab,kw OR EPAS1:ti,ab,kw OR FH:ti,ab,kw OR TMEM127:ti,ab,kw OR HIF2A:ti,ab,kw OR HRAS:ti,ab,kw OR KIF1B*:ti,ab,kw OR PHD2:ti,ab,kw OR neurofibromato*:ti,ab,kw OR [mh ”Neurofibromatosis 1”] OR von Recklinghausen*:ti,ab,kw OR [mh ”Multiple endocrine neoplasia”] OR ”Multiple endocrine neoplasia”:ti,ab,kw OR [mh ”von Hippel-Lindau Disease”] OR von Hippel-Lindau*:ti,ab,kw) |
| #4 (#2 OR #3) |
| #3 ((adren*:ti,ab,kw OR organ*:ti,ab,kw OR cortic*:ti,ab,kw) AND (preserv*:ti,ab,kw OR sparing*:ti,ab,kw)) |
| #2 (partial*:ti,ab,kw OR subtotal*:ti,ab,kw) |
| #1 (pheochromocytom*:ti,ab,kw OR phaeochromocytom*:ti,ab,kw OR pheochromoblastom*:ti,ab,kw OR phaeochromoblastom*:ti,ab,kw OR paragangli*:ti,ab,kw OR PPGL:ti,ab,kw OR PCC:ti,ab,kw OR “Adrenal Gland Neoplasms”:ti,ab,kw OR “Adrenal Gland Tumor”:ti,ab,kw OR “Adrenal Medulla Tumor”:ti,ab,kw OR [mh “Adrenal Gland Neoplasms”] OR [mh “paraganglioma”]) |
| **Searches of clinical trials registers (ClinicalTrials.gov, European Trials Register, WHO International Trials Registry Platform)**  We searched registers using terms from Group 1 and Group 2 combined with ‘AND’ operator  **ClinicalTrials.gov, European Trials Register, WHO International Trials Registry Platform** |
| \| Pheochromocytoma \| Bilateral \| \| --- \| --- \| \| Paraganglioma \| Hereditary \| \| PPGL \| Removal \| \|  \| Resection \| \|  \| Surgery \| \|  \| Adrenalectomy \| |

**Supplementary Table 2** Risk-of-bias assessment of the included studies using the The Risk Of Bias In Non-randomized Studies of Interventions (ROBINS-I) tool

|  |  |  |  |  |  |  |  |  |  |  |
| --- | --- | --- | --- | --- | --- | --- | --- | --- | --- | --- |
|  | **Study** | Bias due to confounding | Bias in selection of participants into the study | Bias in classification of intervention | Bias due to deviations from intended interventions | Bias due to missing data | Bias in measurement of outcomes | Bias in selection of the reported result | **Overall risk of bias** |  |
|  | Asari 2006 | 3 | 1 | 3 | 2 | 1 | 2 | 2 | **Serious** **Risk of Bias** |  |
|  | Baghai 2002 | 3 | 2 | 3 | 2 | 3 | 2 | 2 | **Serious Risk of Bias** |  |
|  | Castillo 2007 | 4 | 2 | 3 | 2 | 3 | 3 | 3 | **Critical Risk of Bias** |  |
|  | Castillo 2011 | 3 | 2 | 2 | 2 | 1 | 2 | 2 | **Serious Risk of Bias** |  |
|  | Castinetti 2014 | 3 | 2 | 2 | 2 | 2 | 1 | 1 | **Serious Risk of Bias** |  |
|  | Castinetti 2019 | 3 | 3 | 2 | 2 | 2 | 2 | 2 | **Serious Risk of Bias** |  |
|  | Goretzki 1996 | 3 | 2 | 1 | 2 | 1 | 2 | 2 | **Serious Risk of Bias** |  |
|  | Grubbs 2013 | 3 | 2 | 3 | 2 | 3 | 2 | 2 | **Serious Risk of Bias** |  |
|  | Iihara 2003 | 3 | 1 | 2 | 2 | 1 | 1 | 2 | **Serious Risk of Bias** |  |
|  | Inabnet 2000 | 3 | 2 | 3 | 2 | 3 | 2 | 3 | **Serious Risk of Bias** |  |
|  | Janetschek 1998 | 3 | 2 | 3 | 2 | 3 | 3 | 2 | **Serious Risk of Bias** |  |
|  | Jansson 2006 | 3 | 2 | 3 | 2 | 2 | 2 | 2 | **Serious Risk of Bias** |  |
|  | Kittah 2020 | 3 | 1 | 2 | 2 | 2 | 2 | 2 | **Serious Risk of Bias** |  |
|  | Lee 1996 | 4 | 3 | 4 | 3 | 2 | 2 | 2 | **Critical Risk of Bias** |  |
|  | Neumann 2019 | 3 | 1 | 2 | 2 | 2 | 3 | 2 | **Serious Risk of Bias** |  |
|  | Nockel 2018 | 4 | 3 | 3 | 2 | 3 | 3 | 2 | **Critical Risk of Bias** |  |
|  | Pugliese 2008 | 3 | 4 | 3 | 2 | 4 | 3 | 3 | **Critical Risk of Bias** |  |
|  | Qi 2013 | 3 | 1 | 2 | 2 | 2 | 2 | 2 | **Serious Risk of Bias** |  |
|  | Rajan 2016 | 3 | 3 | 3 | 2 | 3 | 3 | 2 | **Serious Risk of Bias** |  |
|  | Sanford 2021 | 3 | 2 | 2 | 2 | 2 | 1 | 2 | **Serious Risk of Bias** |  |
|  | Scholten 2011 | 3 | 1 | 2 | 2 | 2 | 2 | 2 | **Serious Risk of Bias** |  |
|  | Simforoosh 2020 | 3 | 2 | 3 | 2 | 3 | 1 | 2 | **Serious Risk of Bias** |  |
|  | Van Heerden 1984 | 3 | 3 | 4 | 3 | 2 | 3 | 2 | **Critical Risk of Bias** |  |
|  | Walz 2006 | 3 | 2 | 3 | 2 | 2 | 3 | 3 | **Serious Risk of Bias** |  |
|  | Yip 2004 | 3 | 2 | 3 | 2 | 2 | 3 | 2 | **Serious Risk of Bias** |  |
|  |  |  |  |  |  |  |  |  |  |  |

**Supplementary Figure 1** Pooled estimates of pheochromocytoma-specific mortality comparing TA vs PA. CI confidence interval, df degrees of freedom

**
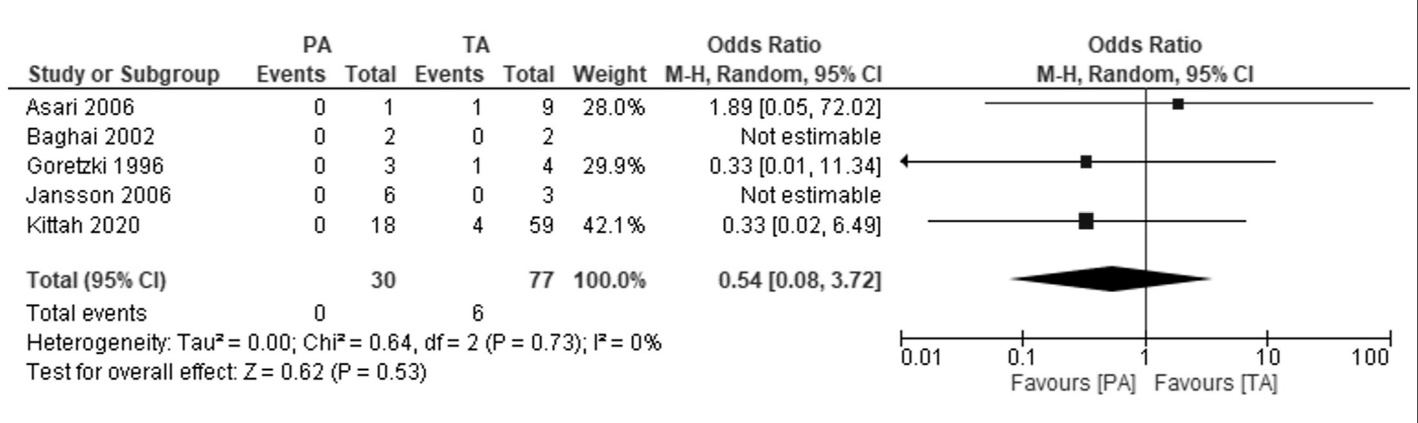
**

**Supplementary Figure 2** Results of sensitivity analyses with omission of one study at a time

1. **Risk of recurrence**


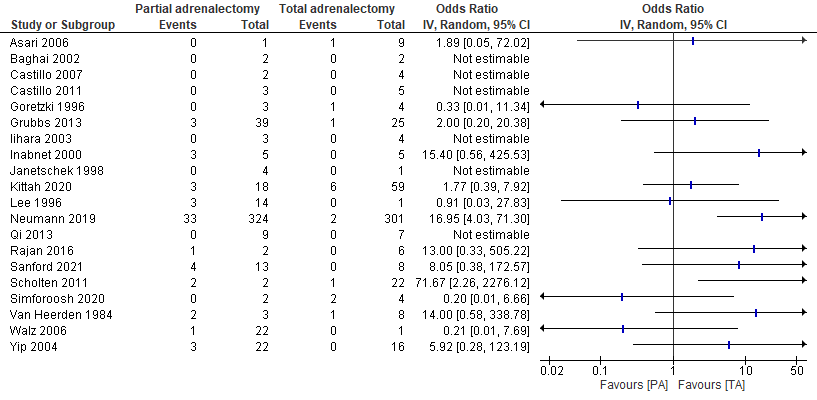


1. **Development of Addisonian crisis**


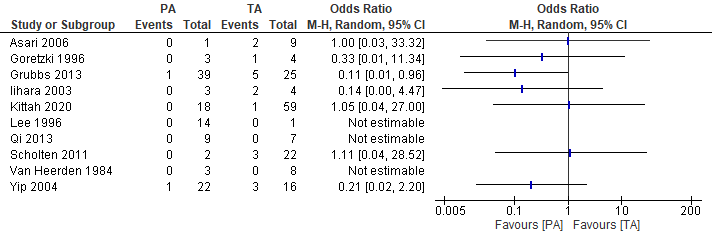


1. **Development of metastatic pheochromocytoma**


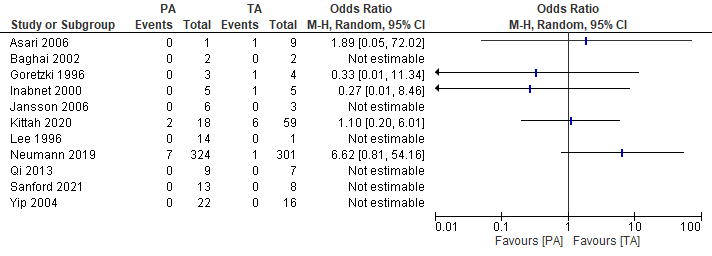


1. **Overall mortality**


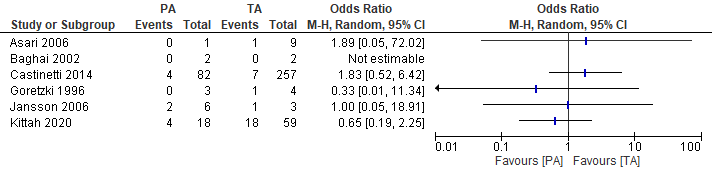


1. **Pheochromocytoma-specific mortality**


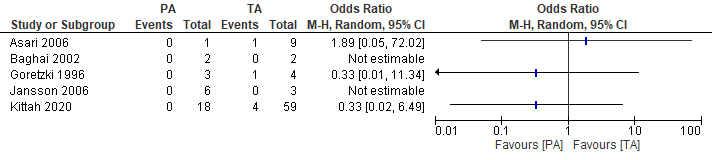

Supplement: Supplementary file 1 [file DataSheet_1.docx]
